# Sin the Supposed Cause of Disease

**Published:** 1871-02

**Authors:** 


Sin the Supposed Cause of Disease.—According to W. A. P. Martin, D.D., L.L.D., Professor of International Law in the University of Pekin, (*American Practitioner*), disease is looked upon in China as a punishment for sin in this or a former life, and, therefore, the spirits who are supposed, by heaven's permission, to inflict these punishments by sending disease must be appeased. The remark is often made by the Chinese that they are expiating their sin in their disease; that is the just retribution of heaven. They are thus ever anxious and ready to resort to the temples to burn incense, to discover the fates, or appease the gods, and evert or remove some malady. The people have more faith in their *gods* than their *doctors*, for although they sometimes seek relief from their native physicians, (if such they deserve to be called), yet they quite as often resort first to the temples. If the patient recovers, it is attributed to the mercy of the god consulted; if he die, it is ascribed to fate. A large and increasing number from all classes, who find no relief from their idols and gods, come at last—often, alas! too late—to the foreign hospital.

